# Investigation of Plasma Treatment on Micro-Injection Moulded Microneedle for Drug Delivery

**DOI:** 10.3390/pharmaceutics7040471

**Published:** 2015-10-30

**Authors:** Karthik Nair, Benjamin Whiteside, Colin Grant, Rajnikant Patel, Cristina Tuinea-Bobe, Keith Norris, Anant Paradkar

**Affiliations:** 1 Centre for Polymer Micro and Nano technology, Interdisciplinary Research Centre, School of Engineering and Informatics, University of Bradford, Bradford, BD7 1DP, UK; E-Mails: B.R.Whiteside@bradford.ac.uk (B.W.); C.Grant@bradford.ac.uk (C.G.); R.Patel@bradford.ac.uk (R.P.); C.Tuinea-Bobe@bradford.ac.uk (C.T.-B.); lavalake22@yahoo.co.uk (K.N.); 2 Centre for Pharmaceutical Engineering Science, School of pharmacy, University of Bradford, Bradford, BD7 1DP, UK; E-Mail: A.Paradkar1@bradford.ac.uk

**Keywords:** plasma treatment, microneedle, micro-injection moulding, atomic force microscope, bovine serum albumin

## Abstract

Plasma technology has been widely used to increase the surface energy of the polymer surfaces for many industrial applications; in particular to increase in wettability. The present work was carried out to investigate how surface modification using plasma treatment modifies the surface energy of micro-injection moulded microneedles and its influence on drug delivery. Microneedles of polyether ether ketone and polycarbonate and have been manufactured using micro-injection moulding and samples from each production batch have been subsequently subjected to a range of plasma treatment. These samples were coated with bovine serum albumin to study the protein adsorption on these treated polymer surfaces. Sample surfaces structures, before and after treatment, were studied using atomic force microscope and surface energies have been obtained using contact angle measurement and calculated using the Owens-Wendt theory. Adsorption performance of bovine serum albumin and release kinetics for each sample set was assessed using a Franz diffusion cell. Results indicate that plasma treatment significantly increases the surface energy and roughness of the microneedles resulting in better adsorption and release of BSA.

## 1. Introduction

Puncturing human skin with hypodermic needles, which is an invasive medical procedure for injecting or collecting biological samples, has been universally accepted for many years [1,2]. Despite the widespread use, hypodermic needle mediated injections cause pain, related infections and sometimes bleeding [3,4]. It is estimated that over 15% of the world’s adult population suffer from trypanophobia (fear of hypodermic needles) [5]. For diabetic patients who are dependent on multiple insulin injections on a daily basis, drug delivery using conventional syringes is the most accepted route [6]. Patch-based transdermal drug delivery offers an alternative way to administer drugs without the drawbacks of the hypodermic needles, but the conventional transdermal drug delivery is limited to those drugs that can diffuse the skin barrier [7]. The outermost layer of the skin stratum corneum provides a significant barrier and only drugs with low molecular weight <500 Da and adequate lipophilicity can be successfully administered. Thus, delivery of hydrophilic drugs and macromolecular agents, like peptides and DNA, are cumbersome [8]. Therefore, facilitation of drug penetration through these biological membranes can only be achieved by reversible disruption of molecular structure of the barrier membranes [8,9]. However, current advances in micromanufacturing technologies offer a number of advantages over hypodermic needles and guided the formulation scientists to design micron-sized needles called microneedles (MNs) [10,11].

MNs consist of a plurality of micro-projections, ranging from 10 to 1800 µm in height and produced from number of different techniques like etching, lithographic, and moulding techniques to produce sharp, high aspect ratio, solid or hollow features in materials, such as plastics, silicon, ceramics, and metals [1,12,13]. MNs, when used to puncture skin will create a transient aqueous transport pathways of micron dimensions and improve permeability [1,14,15].

The emergence of microneedle technologies offers a route for a pain free, straightforward and efficient way of transdermal drug delivery, but technological barriers still exist which pose significant challenges for manufacture of MN systems with high volume outputs at low cost. The main aim of this research was to develop new ways for microneedle manufacture primarily using micro-injection moulding processes with high performance engineering thermoplastics.

Despite the extraordinary versatility of most polymers for solid MN manufacturing, there is one property that markedly reduces its application: a highly hydrophobic surface [16,17,18]. This property makes it difficult to wet and coat the polymeric microneedle surfaces with proteins, vaccines and active pharmaceutical ingredients. These problems arise because the plastic surfaces have relatively poor wetting and adhesion properties, which are due to their low surface energy (absence of polar surface groups) [19,20]. Hence, surface modification of polymeric materials plays an important role to improve surface properties such as wetting and adhesion for coatings, inking and printing processes, biomaterials, and active pharmaceutical ingredients. A well-documented solution for this problem is the use of surface modification of polymer surfaces to increase surface energy.

Various other modification techniques such as, UV irradiation, laser, chemical oxidation, flame and grafting have attracted much attention [21]. Plasma methods are more attractive for many reason like they restrict the surface modifications to depth of several nanometers of the specimen while maintaining its bulk properties, desired surface polarities can be achieved, their low temperature avoids sample destruction [22]. The plasma used for these applications, although not fully ionized, are composed of ions, free electrons, photons, neutral atoms and molecules in ground and excited electronic states [23]. Each of these components has the potential for interaction with surfaces with which they come in contact. The surface wetting and adhesion properties of plasma modified polymers are the subject of many studies and much work has been devoted to the enhancement of polymer–polymer, metal–polymer adhesion, as well as dyeing and printing on the polymers [24]. A good understanding of the surface properties of a solid surface may be obtained relatively inexpensively from the measurement of surface contact angle [25]. The surface free energy of a solid is an important parameter, playing a vital role in the phenomena that occurs at solid–liquid and solid–gas interfaces. Hence, a knowledge of this parameter is useful in studies of adsorption and wettability processes, which play important roles in coated drug delivery using polymeric microneedle devices. Measurement of the contact angle of the liquid with the solid surface permits a rapid and qualitative evaluation of surface free energy of the polymers [26].

During the plasma treatment the surface of the polymer is activated, which brings chain session of the existing groups on the surface and creates new functional groups like hydroxyl, carboxyl, ether and carbonyl which significantly increase the surface free energy. In the recent years there has been a great deal of research carried out on plasma methods to modify polymer surfaces for adhesion for medical implant applications [27,28]. The role that oxygen plasma treatment of injection moulded polymer surfaces can have influence in protein adhesion for drug delivery has yet to be determined in significant detail. Hence, in this paper, the possibility of using plasma treatment to improve drug adsorption and its release from moulded polymer MNs are studied.

## 2. Experimental Section

### 2.1. Manufacture of Microneedles and Flat Polymer Samples for Surface Evaluation

The moulding of the specimens was performed on a Battenfeld Micro-Power 15 (Wittman Battenfeld Group, Vienna, Austria) micro-injection moulding machine, using the parameters mentioned in the Table 1.

**Table 1:** Injection moulding parameters.

Parameter	PEEK	PC
Melt temperature (°C)	420	320
Injection velocity (mm/s)	800	500
Clamping force (kN)	150	100
Time of holding pressure (s)	5	3
Mould base temperature (°C)	200	120

**Figure 1:**
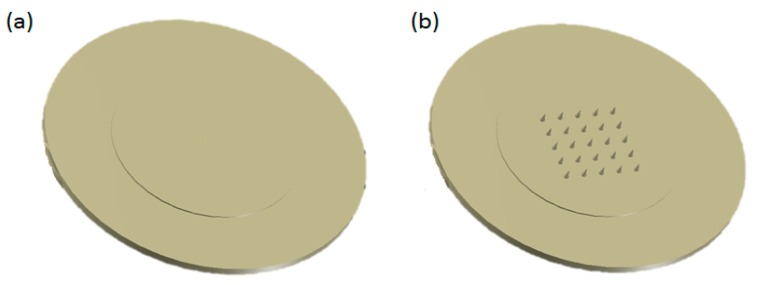
Micro-injection moulded samples for (**a**) flat polymer for surface analysis and (**b**) moulded MN array for dip coating.

The mould for MN manufacture was used to produce specimen for surface evaluation but instead of using the MN insert, a flat mould insert and a polished sapphire window (1.5 nm roughness) was used on the moving side of the mould. Two candidate materials, Polycarbonate (PC-Lexan HPX8REU) and Polyether ether ketone (PEEK, Optima LT-3), were selected for micro-injection moulding. Lexan is manufactured by Sabic Innovative Plastics while PEEK LT-3 is FDA approved semi crystalline biomaterial launched by Victrex (Invibio Inc., Lancashire, UK). Moulded specimens of each material were of 17 mm diameter and 0.5 mm thickness, which was ideal for the surface characterisation. Figure 1a shows the top view of the moulded specimen used for surface analysis and Figure 1b shows the schematic of the 5 × 5 microneedle array. Microneedles with a shaft length of 550 and 300 µm base width and a tip radius of 30 µm in width was manufactured by micro-injection moulding.

### 2.2. Oxygen Plasma Treatment

All the micro-injection moulded samples of PEEK and PC were rinsed with deionised water and air dried before the plasma treatment. The plasma treatment system (nano series plasma surface machine by Diener electronic, Jettingen, Germany) with a generator that works with radio-frequency signal of 40 kHz was used for the study. Samples of moulded specimens, which needed plasma activation, were placed in the plasma chamber and the vacuum pump was utilised to reduce the pressure to 0.1 mbar and at that pressure of the process gas (*i.e.*, oxygen 99% of purity) was fed into the chamber. When this working pressure was achieved the generator was switched on and process gas in the recipient was ionised. The plasma treatment time was varied between 10, 20, 30, 40, 50, and 60 min.

### 2.3. Contact Angle and Surface Energy Analysis

Flat moulded samples of PEEK and PC were washed and subsequent experimental procedures were performed using FTA188 video tensiometer (First Ten Angstroms, Inc., Portsmouth, VA, USA). Static contact angle studies were performed using various sets of probe liquids (*n*-octanol, *n*-hexane, *n*-octane, *n*-heptane, cyclohexane, toluene, benzyl alcohol, ethylene glycol, glycerol and double distilled water). Practically, droplets (~0.7l of probe liquids) were released on the polymer surface and an optical system was used to analyse the drop profile. The contact angle (θ) value was calculated using image analysis FTA32 software. Surface free energy (σ***_s_***) and its polar (σ***_s_^P^***) and dispersion (σ***_s_^D^***) components of the samples were determined from sets of contact angles made by the probe liquids according to Owens/Wendt equation.

(1) $$\frac{\sigma_{L}\left(cos\theta +1\right)}{2\left(\;\sigma_{L\;}^{D}\;\right){\;}^{1/2}}=\;\left(\;\sigma_{S\;}^{P}\;\right){\;}^{1/2}\;\frac{\left(\;\sigma_{L\;}^{P}\;\right){\;}^{1/2}}{\left(\;\sigma_{L\;}^{D}\;\right){\;}^{1/2}}+\left(\;\sigma_{S}^{D}\;\right){\;}^{1/2}$$

where σ***_L_*** *=* surface tension of the wetting liquid, σ***_L_^D^*** *=* dispersive component of surface tension of the liquid, σ***_L_^P^*** *=* polar component of surface tension of the liquid, σ***_s_*** *=* overall surface energy of the test solid, σ***_s_^D^*** *=* dispersive component of the surface energy of the solid, σ***_s_^P^*** *=* polar component of the surface energy of the solid and σ***_sL_*** *=* interfacial tension between solid and liquid.

### 2.4. AFM Analysis

Atomic force microscope was used to examine the changes in the polymer surface before and after the plasma treatment process. All AFM scans were made using an MFP-3D scanner from Asylum Research, Santa Barbara, CA, USA. Silicon nitride cantilever tips (Applied Nanostructures, Santa Clara, CA, USA) with a tip radius of 15 nm and spring constant of 0.3 Nm were used. The scans were made on 10 µm × 10 µm scale and repeated five different times on five different area of the polymer film at a resolution of 512 × 512 pixels. Images were interpreted using integrated MFP-3D™ Igor software (Asylum Research, Santa Barbara, CA, USA). Errors in the piezo-linearity were corrected for by using zero and first order flattening. The scans were made on 10 µm × 10 µm scale and repeated five different times on five different area of the flat polymer surface.

### 2.5. Surface Morphology after Protein Adsorption

Protein solution of 50 µg/mL was prepared with lyophilized powder of Bovine Serum Albumin (BSA) (Sigma–Aldrich, Dorset, UK) in Phosphate Buffer Solution (PBS) of pH 7.4. Plasma treated and untreated polymer samples were immersed in 5 mL protein solution and allowed to incubate at 37 °C. After 1 h samples were removed from the incubator and rinsed with Milli-Q water to remove unbounded protein. All the polymer samples were then allowed to dry at room temperature and subsequently analysed by AFM to understand the protein adsorption before and after plasma treatment. The scans were made on 1.5 µm × 1.5 µm scale and repeated five different times on five different area of the polymer surface.

### 2.6. Coating Arrays of Microneedles

**Figure 2:**
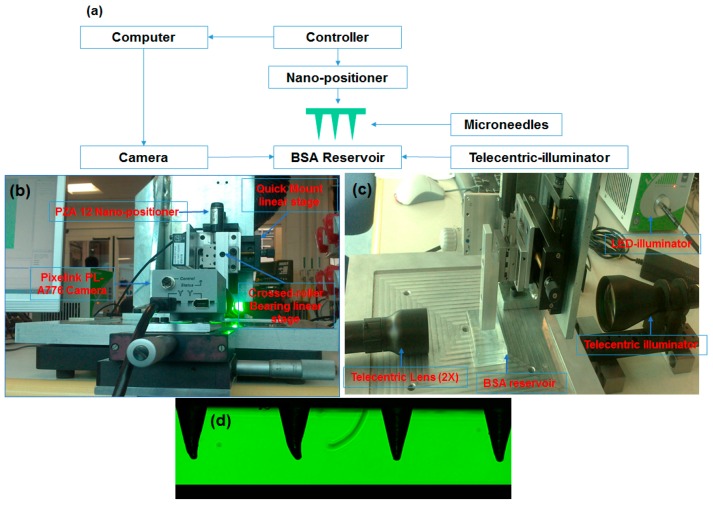
(**a**) Schematic of the nano-positioner dip coating set up; (**b**) photograph of dip coating apparatus showing individual components; (**c**) side view showing the lens and the BSA reservoir; (**d**) microneedle array as seen from the software before dip coating.

Rows of solid micro-injection moulded MNs were dip coated using an in-house designed nano-positioning coating device. The coating device consisted of two parts: (1) the micro-positioning dip coater and (2) the coating solution reservoir, a schematic of the dip coater is shown in Figure 2a.

*Nano-positioning dip coater*: it was designed to restrict access of the coating liquid only to the microneedle shaft to prevent contamination of the base, to provide sufficient time to the microneedle inside the BSA reservoir and to slowly retract the MNs from the reservoir. To enable three-dimensional alignment and dipping of microneedle arrays into the reservoir, a piezoelectric nano-positioner (PZA12) were assembled on a cross-roller linear stage, which hold the microneedle arrays (Figure 2b). To view the whole dipping process a Pixelink PL-A776 3.1 mega pixel CMOS colour camera coupled with a 2× telecentric lens and a LED fibre optic telecentric illuminator were used. The coating was performed automatically using software Lab View (National Instruments, Austin, TX, USA).

*Coating solution reservoir*: The reservoir was custom made of specific dimension from quartz. The BSA solution used for dip coating was of a concentration of 10 mg/mL solution.

### 2.7. Analysis of BSA in Solution

BSA analysis was performed using HPLC method with a gradient system that consists of 0.1% trifluoroacetic acid in water (A) and 0.08% trifluoroacetic acid in acetonitrile (B) with initial A/B ratio of 80/20, which was changed linearly to the final ratio of 35/65 (A/B) within 15 min. The flow rate was 1 mL/min and BSA separation was carried out using a Symmetry 300 C4 protein analysis column (50 mm× 4.6 mm; 5 µm particle size, Waters, Milford, MA, USA). A UV detector (Waters, USA) at wavelength of 220 nm was used for BSA detection. The injection was made using a loop injector equipped by a 50 µL loop. A stock solution of BSA of concentration 100 µg/mL was prepared in phosphate buffer (pH = 7.4) and various concentrations of 1, 2, 4, 8, 10 and 25 µg/mL were prepared by serial diluting the stock solution. These samples were then injected into the HPLC and then linear regression analysis was out on known concentration and corresponding peak heights. The regression coefficient (*r*) slope and intercept of the resulting calibration curves were determined.

### 2.8. Drug Release Studies from Microneedles and in Vitro Diffusion Studies

**Figure 3:**
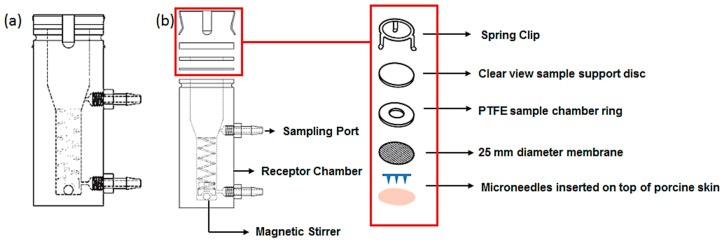
Schematic of the Franz diffusion experimental setup: (**a**) vertical diffusion cell; (**b**) exploded view of the cell and sample holder (adapted from www.copley.com).

Passive diffusion of BSA (a water soluble protein with MW 69 KDa) from the polymeric microneedle arrays across dermatomed abdominal neonatal porcine skin was investigated *in vitro* using Franz diffusion cells (Copley, Nottingham, UK). Neonatal porcine skin samples were shaved and excess fat was removed and pre-equilibrated in phosphate buffer (pH 7.4) for 2 h before the experiments. BSA coated MNs were inserted into the circular specimen of skin which was secured to the donor compartment of the Franz diffusion cell. It was then placed on top of the receptor cell, which was filled with 7 mL of PBS. This complete unit was then mounted on to the Franz cell with synchronously stirred using magnetic teflon stir bars at constant speed of 400 rpm and thermostabilised at 37 ± 1 °C. A schematic of the experimental setup is shown in Figure 3. At different time points, aliquots from the receptor medium was withdrawn from the sampling arm and replaced with fresh preheated phosphate buffer pH 7.4 (at 37 °C). The sink condition was maintained throughout the experiment and concentration of BSA in the receptor medium was determined using HPLC. The cumulative amount of BSA permeated was calculated using PCP disso Software (Pune college of Pharmacy, Pune, India).

### 2.9. Quantification of the Residual BSA from the MN Shaft after Insertion

Untreated and plasma treated PEEK and PC microneedles were dip coated with 10 mg/mL of BSA for 10 min. These MNs were then inserted into the porcine skin using the thumb pressure and removed after 15 min. The MNs after the insertion were transferred to a vial containing 2.5 mL of PBS solution and ultra-sonicated to remove the residual BSA on the MNs and the amount of the residual BSA was then quantified using the HPLC.

## 3. Results and Discussion

### 3.1. Plasma Treatment, Contact Angle and Surface Energy

**Figure 4:**
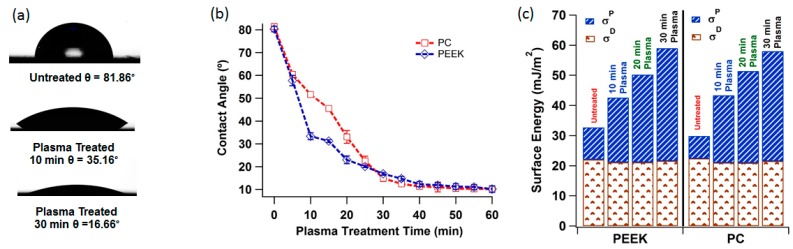
(**a**) Water contact angle PEEK before and after 10 and 30 min of oxygen plasma treatment; (**b**) graph showing water contact angle with plasma treatment time for PEEK and PC; (**c**) bar graph showing the polar (σ^P^) and dispersive (σ^D^) component of the surface energy of the three polymers before and after plasma treatment calculated using Owens/Wendt theory.

As expected, the water contact angles made on untreated PEEK (Figure 4a) are larger (above 80°) because of the low-energy and hydrophobic nature of the polymer surfaces. The surface used for testing contact angle was made by micro-injection moulding having a sapphire window on one side of the mould resulting a very smooth surface. The total surface free energy (polar and dispersive component) of the polymer is shown in the Figure 4c. The values of polar and dispersive component of the polymer according Owens/Wendt model equation shows that molecular interactions are mainly dispersive. The wetting behaviour of the polymer samples were dramatically changed after oxygen plasma treatment. It was observed that for both the polymers oxygen plasma treatment lowers the contact angle as compared to the untreated samples. Figure 4b shows the water contact angle values for untreated and plasma treated polymer samples. This was expected, since the plasma activation process more oxygen-containing functional groups of the polymers are generated on the polymer surface caused by the reactions between the polymer and active species from the oxygen plasma thus increasing the hydrophilicity. Another important factor which increases the wettability is the surface modification mechanism by UV radiation emitted by plasma, the exposure to the oxygen plasma discharge is sufficient to break chemical bonds (C–C, C–H), and leaves free radicals at or near the surface [29]. These radicals can react only with other surface radicals or by chain transfer reaction [30,31]. If the polymer surface is flexible, or if the radicals can migrate along it, then recombination, unsaturation or cross-linking can occur [32]. Moreover, the plasma removes low molecular weight species or converts them to high molecular weight species by cross-linking reaction [33]. In summation, the surface of the polymer is activated during plasma treatment, which brings about the chain session of the existing groups on the surface of the polymer and creates new functional groups, such as –OH and –OOH.

### 3.2. Plasma Treatment and Surface Topography

The effect of plasma treatment depends on internal and external parameters like type of plasma (DC, RF, or microwave), the discharge power density, pressure, and flow rate of the gas or gas mixture and exposure time [34]. Oxygen plasma is used to increase polar functional groups (hydroxyl, carboxyl, ether, carbonyl, *etc.*) [27,35], which can successfully increase the surface free energy of the polymer [31]. In the recent years, the surface treatment of polymers has been performed by many researchers to make them suitable for adhesion [19,36,37,38].

**Figure 5:**
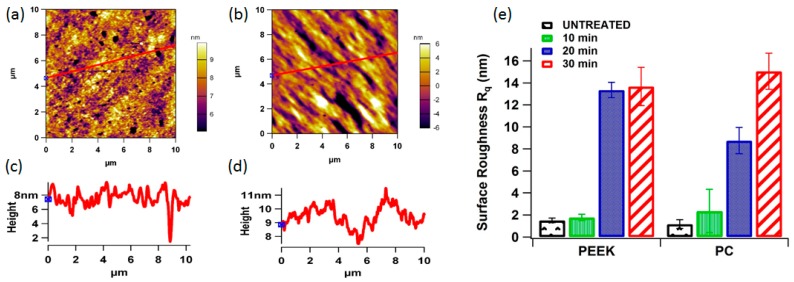
Representative 2D non-contact mode AFM images (10 µm × 10 µm) for untreated (**a**) PEEK and (**b**) PC; (**c**,**d**) surface roughness line profiles for untreated PEEK and PC; (**e**) bar graph showing the surface roughness of each polymer *vs.* plasma treatment time.

The morphologies of the two experimental untreated polymeric surfaces are shown in Figure 5a,b, as viewed by the AFM. This is a local probe technique that reveals local structural features, which are not necessarily representative for the whole sample surface. Due to this reason, an appropriate way to record and process many images from different areas of the surface was used. This furnishes local structure information with high resolution, but also enables to determine a mean behaviour. The representative AFM images of untreated polymer surfaces and scans were made on 10 µm × 10 µm scale and repeated five times and average surface roughness was used. All the evaluations that are described in the following have, thus been carried out quantitatively and averaged over a large number of pictures recorded in different areas of the samples. The mean surface roughness *R*_q_ of untreated PEEK and PC were 1.51 and 1.61 nm, respectively. This confirms that the surfaces were relatively smooth for all polymers, which is important for surface characterisation.

**Figure 6:**
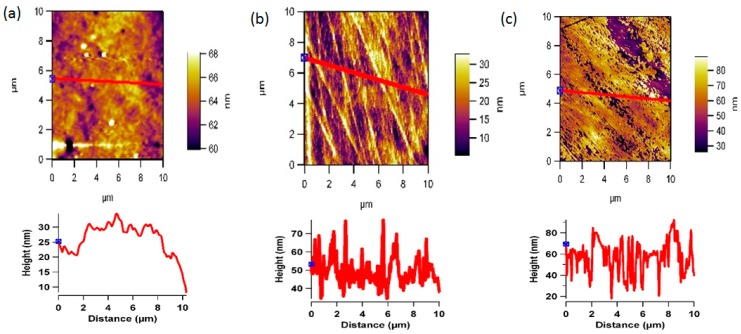
Representative 2D non-contact mode AFM images (10 µm × 10 µm) and line profiles for PEEK after (**a**) 10, (**b**) 20, and (**c**) 30 min of oxygen plasma treatment.

**Figure 7:**
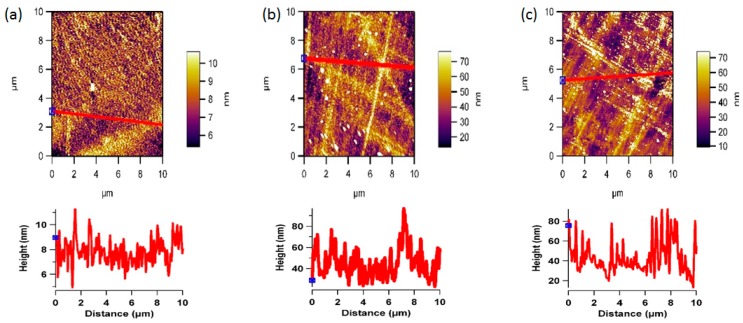
Representative 2D non-contact mode AFM images (10 µm × 10 µm) and line profiles for PC after (**a**) 10, (**b**) 20, and (**c**) 30 min of oxygen plasma treatment.

The AFM images Figure 6 and Figure 7 reveals the surface morphology and the line profiles of the PEEK and PC surfaces after plasma treatment for 10, 20, and 30 min respectively. It is well known that the wettability of a polymer surface is affected by the surface morphology, especially the surface roughness. AFM scans to examine the changes in the surface morphology induced before and after oxygen plasma treatment was performed. After the plasma treatment for 30 min showed the presence of aggregate structure (sign of patchiness or inhomogeneities) with various sizes on the surface. The different sizes of the aggregate indicate the uneven effect of the surface etching by plasma treatment. The plasma treatment time was optimised by comparing the contact angle and AFM data. The AFM scans showed that after 30 min, further exposure can simply etch away the material that was previously modified by the plasma. This can also be the reason for minima and maxima properties as function of time, and highlights the importance of optimising the treatment period and to optimise the plasma treatment time. Contact angle *vs.* plasma treatment time was measured and found out that after 30 min of plasma there is no significant decrease in the contact angle, as shown in Figure 4b.

The plasma treated surfaces of both the polymers also showed the formation of the pit-like structures, probably because of sputtering effects of plasma, which was predominantly observed on the PC surface. Changes in the surface roughness of the oxygen plasma treated polymer samples were quantified by root-mean square values to highlight the surface modifications due to oxygen plasma treatment at 30 min. Figure 4c shows average roughness values of untreated and plasma treated samples. The average surface roughness increases from 1.51 to 1.61 nm for the untreated PEEK and PC, reaching a value of 13.34 and 15.48 nm after 30 min of plasma treatment respectively. This indicates that PC surface roughness has slightly increased more than that of PEEK suggesting PEEK to be more resistant to plasma treatment as compared to PC [39]. These results are indicative that the effects of oxygen plasma treatment on the polymer surface observed by AFM are due to plasma bombardment of the surface and scission of the polymeric chains, which in turn rearrange themselves to give rise to a new surface morphology [33].

### 3.3. Plasma Treatment and Protein Adsorption

**Figure 8:**
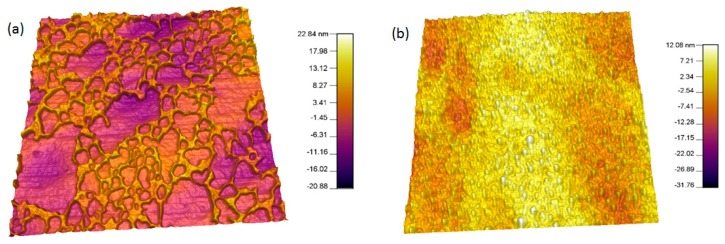
(**a**) Representative 3D Tapping mode AFM scans of untreated PEEK + BSA showing the how the BSA is adsorbed on a hydrophobic polymer surface; (**b**) plasma treated PEEK + BSA showing the how the BSA is adsorbed on a surface modified polymer surface.

Protein adsorption studies to understand the adhesion of BSA on to both treated and untreated polymer surfaces were conducted. The 3D AFM image (Figure 8a,b) of both the untreated and treated PEEK samples gives a clear understanding of the BSA adsorption at the polymer surfaces.

In the case of untreated samples, a patchy pattern of BSA was observed, whereas for plasma treated, the BSA was uniformly spread over the entire polymer surface, indicating better adsorption. A protein distribution analysis done by the MFP-3D™ Igor software confirmed that 17%–20% of the polymer surface was coated with BSA in the case of untreated specimen and thickness of the BSA coat was found to be 14.33 ± 3.2 nm.

### 3.4. Analysis of BSA

A high performance liquid chromatography method was used to quantify the BSA adsorbed on the polymer surfaces. A calibration curve was obtained for BSA standard solutions of (1, 2, 4, 8, 10 and 25 µg/mL) (*r*^2^ = 0.9985).

**Table 2:** Accuracy and precision of the HPLC method (*n* = 3).

Selected concentration (µg/mL)	Mean concentration found (µg/mL)	SD	Accuracy (%)
**Inter-day**			
1 (low)	0.98	0.02	98.62
50 (medium)	46.45	4.67	92.91
300 (high)	297.57	1.92	99.19
**Intra-day**			
1 (low)	1.034	0.11	96.66
50 (medium)	48.91	6.84	97.83
300 (high)	295.13	7.41	98.37

Table 2 describes the accuracy and precision of the inter-day and intra-day analyses of three different concentration (low, medium, and high) of BSA. The values obtained from the assay procedure showed good reproducibility with similar intra-day and inter-day results. A calibration curve was used to determine the unknown concentration of BSA adsorbed on to the surface modified polymer samples.

### 3.5. Quantification of Residual BSA from the MN Shaft after Insertion and in Vitro Delivery of BSA across the Neonatal Porcine Skin

**Figure 9:**
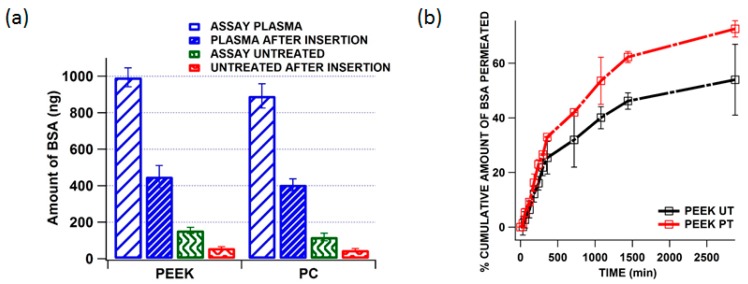
(**a**) Comparison of amount of BSA coated on to the plasma and untreated PEEK and PC microneedles and residual BSA from the needle after insertion; (**b**) comparative permeation profiles of BSA across neonatal porcine skin when released from untreated and plasma treated MN (PEEK). Means (±SD), *n* ˃ 3.

Solid MNs are generally used for proteins and vaccine delivery and the efficiency of these MNs depends on how quick the coated formulation is released from MNs to the skin. In this study we quantify and compare the residual BSA that is left in the untreated and plasma treated MN shaft after insertion onto porcine skin for 15 min. Figure 9a shows the amount of BSA loaded (assay) and residual (after insertion) to untreated and treated MNs of PEEK and PC. It is evident that the plasma treated MNs could load up to 1 µg of BSA and the residual amount after insertion was found to be 428 ng that means 572 ng of BSA was been delivered into the skin in first 15 min whereas in the case of untreated MNs could only load 183 ng and the residual amount after insertion was found to be 64 ng which means 119 ng of BSA was delivered into skin from the untreated MNs. From this it was observed that the amount of BSA loaded was more in the case of plasma treated MNs and just over half the amount was released but the in the case of the untreated MNs even though the initial loading was 5.7 times over than the plasma treated MNs the proportion of the original amount which was delivered to the skin was slightly higher (65% *vs.* 57.2%). This is because plasma along with increasing the surface energy of the polymer surfaces it also increases the adhesion of BSA to the MN shafts resulting in a reduced release rate.

Figure 9b shows the comparative passive permeation of BSA across neonatal porcine skin when released from untreated and plasma treated PEEK MNs. The results indicate that the BSA delivery from the plasma treated MNs were found to be more efficient than that from untreated MNs. Although plasma treated MNs were able to receive a higher amount of the BSA during coating than untreated samples, the total amount of the BSA that could be delivered was limited because of relatively small surface area of the MNs. Using BSA as a model drug a maximum of 1 µg of BSA (totally loading amount) was coated on 25 plasma treated MNs (550 µm long and 300 µm wide). The plasma treated and untreated MNs showed similar release for the initial 6 h and during the 24 h it was found that more than 60% of BSA permeated from plasma treated MNs. This is because plasma treatment increases the surface energy and enhances BSA adsorption onto the needle shaft.

## 4. Conclusions

This study has demonstrated, for the first time use of plasma treatment in increasing the adsorption of BSA onto the MNs shaft for enhanced transdermal delivery. Modification created by plasma changed the surface of the microneedles both chemically and morphologically. This resulted in the increase of overall surface energy and wettability of the MN surface leading to increase adsorption of BSA compared to untreated MNs.
